# Dipeptidyl Peptidase 4 Restoration Facilitates Antitumor Immunity in KRAS-LKB1–Mutant Lung Cancer

**DOI:** 10.1158/2767-9764.CRC-25-0199

**Published:** 2025-12-17

**Authors:** Toshiyuki Tenma, Ryohei Yoshida, Hiraku Yanada, Kiichi Nitanai, Toshihiro Nagato, Kyohei Oyama, Nobunari Sasaki, Mizuki Homme, Chie Mori, Takayuki Ohkuri, Yusuke Ono, Shoichiro Tange, Yoshinori Minami, Hiroya Kobayashi, Yusuke Mizukami, David A. Barbie, Shunsuke Kitajima, Takaaki Sasaki

**Affiliations:** 1 Division of Respiratory Medicine and Neurology, Department of Internal Medicine, Asahikawa Medical University, Asahikawa, Japan.; 2 Clinical Research Center, Keiyukai Yoshida Hospital, Asahikawa, Japan.; 3 Department of Pathology, Asahikawa Medical University, Asahikawa, Japan.; 4 Department of Cardiac Surgery, Asahikawa Medical University, Asahikawa, Japan.; 5 Project for Cancer Immunotherapy Development, Cancer Precision Medicine Center, Japanese Foundation for Cancer Research, Tokyo, Japan.; 6 Institute of Biomedical Research, Sapporo Higashi Tokushukai Hospital, Sapporo, Japan.; 7 Department of Medical Genome Sciences, Cancer Research Institute, Sapporo Medical University School of Medicine, Sapporo, Japan.; 8 Division of Gastroenterology, Department of Medicine, Asahikawa Medical University, Asahikawa, Japan.; 9 Department of Medical Oncology, Dana-Farber Cancer Institute, Boston, Massachusetts.

## Abstract

KRAS proto-oncogene, GTPase (*KRAS*)-liver kinase B1 (*LKB1*)–mutant (KL) non–small cell lung cancer (NSCLC), characterized by a profoundly immunosuppressive tumor microenvironment (TME), is highly resistant to immune checkpoint inhibitors. Despite their high tumor mutation burden, KL tumors exhibit low expression of PD-L1, reduced immune cell infiltration, and suppressed immune signaling pathways. Systematic genome analyses revealed that LKB1 loss suppresses dipeptidyl peptidase 4 (DPP4) expression and activity in *KRAS*-mutant lung cancer. The therapeutic potential of restoring DPP4 function to improve the immune response in KL lung cancer was evaluated using patient-derived tumor samples and syngeneic mouse models. Restoration of DPP4 expression reprogrammed the TME and significantly increased immune-related gene signatures, including those involved in T-cell migration and NK-cell activation. Restoration of DPP4 expression in three-dimensional microfluidic models enhanced NK-cell chemotaxis and spheroid-targeting activity. Furthermore, DPP4 restoration was synergized with anti–PD-1 therapy to achieve significant tumor regression in syngeneic KL murine models. These findings suggest that LKB1 loss suppresses DPP4 expression, contributing to the immunosuppressive characteristics of the TME in KL-NSCLC cells, whereas restoring DPP4 expression promotes NK-cell recruitment, facilitates immune activation, and enhances the effects of anti–PD-1 therapy. These results suggest that DPP4 is a key immune modulator and a promising therapeutic target, providing a novel strategy to overcome immune resistance and improve immunotherapy outcomes in this challenging subset of lung cancer.

**Significance:**

LKB1 loss suppresses DPP4 expression in KL-NSCLC; however, restoring DPP4 expression *in vitro* promotes NK-cell recruitment, mitigating the immunosuppressive TME and enhancing the efficacy of anti–PD-1 therapy in KL models.

## Introduction

Immunotherapy, particularly with immune checkpoint inhibitors (ICI), has revolutionized the treatment landscape of non–small cell lung cancer (NSCLC), offering significant survival benefits in many cases (1). However, most patients with cancer fail to respond durably to ICIs, and the emergence of resistance to ICIs poses a major challenge. Several cancer resistance mechanisms have been identified, including a low tumor mutation burden, loss of antigen presentation via MHC class I, and impaired or ineffective immune cell infiltration (2).

KRAS proto-oncogene, GTPase (*KRAS*)–mutant NSCLCs present an additional layer of complexity as their response to ICIs is highly variable and largely influenced by cooccurring genetic alterations (3). Specifically, *KRAS*-mutant NSCLCs with concurrent liver kinase B1 [*LKB1*; also known as serine/threonine kinase 11 (*STK11*)] mutations (KL) exhibit marked resistance to ICIs, in contrast to those with tumor protein p53 (*TP53*) co-mutations (KP), which often respond favorably (4). KL tumors demonstrate a profound immunosuppressive phenotype, including low PD-L1 expression, impaired immune cell infiltration, and poor responsiveness to ICIs (5). Although KL tumors often display a high tumor mutation burden, which is generally predictive of favorable ICI responses, this paradoxical resistance persists. This highlights the crucial role of co-mutations in shaping the tumor–immune microenvironment and influencing the efficacy of ICIs. In this study, KL refers only to tumors harboring KRAS mutations and genetic alterations in STK11 that result in the loss of LKB1 function, irrespective of the presence or absence of KEAP1 alterations (6).

We previously reported that the loss of LKB1 in KL tumors suppresses key immune pathways, including the cyclic GMP–AMP synthase stimulator of interferon genes (STING) signaling axis, which plays a central role in innate immune activation (7). Epigenetic silencing of STING further exacerbates immune evasion, creating a tumor microenvironment (TME) that is refractory to immune surveillance and therapy. Although preclinical studies using STING agonists or epigenetic modulators have shown promise in reactivating immune responses, KL tumors continue to pose significant therapeutic challenges (8). This highlights the urgent need for innovative strategies to address the immunosuppressive microenvironmental characteristics of this subset of NSCLC.

In this regard, dipeptidyl peptidase 4 (DPP4; also known as CD26), an enzyme that regulates immune responses and maintains cellular homeostasis, is a potential therapeutic target in *KRAS*-mutant NSCLC. Although DPP4 has been implicated in other malignancies, its specific role in *KRAS*-mutant lung cancer and association with ICI resistance remain unknown (9). Preliminary findings suggest that the loss of LKB1 suppresses DPP4 expression and activity, which may further exacerbate the immunosuppressive phenotype of KL tumors (9, 10).

In this study, we aimed to evaluate the therapeutic potential of restoring DPP4 function to improve the immune response in KL lung cancer using genomic analyses, patient-derived tumor samples, and syngeneic mouse models. Our findings provide insights into the potential of DPP4 as a target for overcoming ICI resistance in this challenging subset of *KRAS*-mutant NSCLC.

## Materials and Methods

### Cell lines

H1355 and H2009 cells were cultured in DMEM (Thermo Fisher Scientific, cat. #11965-118) supplemented with 10% FBS (Biosera, cat. #FB-1003-500) and 0.5% penicillin–streptomycin (Gemini Bio-Products, cat. #400-109). A427, A549, H23, HCC44, H647, H1944, H2122, H358, H441, H1792, H2291, Calu1, H1793, MC38, and 393P cells were cultured in RPMI-1640 (Thermo Fisher Scientific, cat. #11875-119) supplemented with 10% FBS and 0.5% penicillin–streptomycin. NK-92 cells were cultured in H5100 medium (STEMCELL Technologies, cat. #05150) supplemented with 200 U/mL recombinant IL2 (PeproTech, cat. #200-02), 12.5% horse serum (Thermo Fisher Scientific, cat. #16050130), and 1% penicillin–streptomycin. A427, A549, H23, HCC44, H1944, H2122, H1792, H1355, H2009, and MC38 cells were originally obtained from the Broad Institute and authenticated by short tandem repeat genotyping. HEK293T, H647, H358, H441, H2291, Calu1, H1793, and NK-92 cells were purchased from ATCC. 393P cells were established from Kras^LA1/+^; p53^R172HΔG^ mice and kindly gifted by Dr. J.M. Kurie (The University of Texas, MD Anderson Cancer Center, Houston, TX). All experiments were performed before reaching 10 passages from the original frozen stocks. *Mycoplasma* infection was regularly checked with the MycoAlert Mycoplasma Detection Kit (Lonza, cat. #LT07-218) according to the manufacturer’s instructions.

### Reagents and treatments

This study used the following reagents: sitagliptin (Selleckchem, cat. #S5079), metformin (Selleckchem, cat. #S5958), and compound C (Merck Millipore, cat. #171260).

### IHC staining

Formalin‐fixed, paraffin‐embedded samples were prepared from biopsy and surgical tissues obtained from patients with *KRAS*-mutant NSCLC at Asahikawa Medical University and cut into 4‐μm‐thick sections. These samples were collected with the approval of the Institutional Review Board at Asahikawa Medical University. The primary antibodies used were monoclonal anti-LKB1 mouse (Abcam, cat. #ab15095, 1:500) and anti-DPP4 rabbit (Abcam, cat. #ab215711, 1:1,000). Formalin‐fixed, paraffin‐embedded specimens were stained with VENTANA BenchMark GX (Roche Diagnostics) using Cell Conditioning 1 buffer (Roche Diagnostics) as the antigen retrieval solution and a VENTANA ultraView Universal DAB Detection Kit (Roche Diagnostics). Representative images were acquired using a BZ-X710 microscope (Keyence). A case was considered LKB1 positive if >10% of the tumor cells were positive for LKB1. The intensity of DPP4 staining was classified as no, weak, moderate, and strong. Moderate and strong staining indicated positive DPP4 expression, whereas no and low staining indicated negative DPP4 expression. Two pathologists independently evaluated all stained sections.

### CRISPR/Cas9 system and lentiviral infection

Target sequences for CRISPR interference were designed using a single-guide RNA (sgRNA) designer (http://portals.broadinstitute.org/gpp/public/analysis-tools/sgrna-design). A nontargeting sgRNA from the Gecko Library v2 was used as the scrambled sgRNA. The sgRNA target sequences are listed in Supplementary Table S1.

HEK293T cells (3 × 10^6^) were plated onto a 60-mm dish and transfected using X-tremeGENE HP DNA Transfection Reagent (Roche, cat. #06366236001) with 1 μg lentivirus-based expression vector together with 1 μg pCMV-dR8.91 and 1 μg pCMV-VSV-G. After incubation for 48 hours, media containing lentivirus particles were collected, passed through a 0.45-μm filter, and concentrated using a Lenti-X Concentrator (Clontech, cat. #631231). hDPP4 and mDPP4 constructs were purchased from VectorBuilder. Virally infected cells were selected using 0.5 to 2 μg/mL of puromycin (pCRISPR-v2 sgRNAs, plx307-NanoLuc, plx307-hDPP4, and plx307-mDPP4) or 1.5 to 8 μg/mL of blasticidin (plx304-GFP, plx304-LUC, and plx304-hLKB1-V5) 24 hours after infection.

### qRT-PCR

RNA was extracted using the RNeasy Mini Kit (cat. #74106). RNA samples (1 μg) were reverse-transcribed using SuperScript III First-Strand Synthesis SuperMix (Thermo Fisher Scientific, cat. #1683483). qRT-PCR was performed using the Power SYBR Green PCR Master Mix (Thermo Fisher Scientific, cat. #4367659), and the primer sequences are listed in Supplementary Table S1.

### Immunoblotting

Cells were lysed in RIPA buffer (Thermo Fisher Scientific, cat. #89900) or NP-40 Cell lysis buffer (Invitrogen, cat. #FNN0021) containing 1× protease inhibitors (Roche, cat. #11-873-580-001) and phosphatase inhibitors (Sigma-Aldrich, cat. #4906837001). Thereafter, lysates were centrifuged at maximum speed for 4.5 minutes at 4°C, and the supernatant was used for subsequent procedures. Western blot analyses were conducted after separating the proteins using SDS-PAGE and transferring them to Immobilon-P membranes (Merck, cat. #ISEQ07850). Immunoblotting was performed according to the manufacturer’s recommendations using the following antibodies: DPP4/CD26 (#67138, Cell Signaling Technology), AMP-activated protein kinase α (AMPKα; #5831, Cell Signaling Technology), phospho-AMPKα (#2535, Cell Signaling Technology), phospho-STAT1 (#9167, Cell Signaling Technology), LKB1 (#13031, Cell Signaling Technology), and β-actin (#4967, Cell Signaling Technology). Anti–rabbit IgG horseradish peroxidase–linked (#7074) secondary antibodies were obtained from Cell Signaling Technology. Imaging of the blots and band quantification were performed using an LAS-500 (Fujifilm).

### Flow cytometry assay

Cells (5 × 10^5^) were resuspended in 100 μL PBS containing 3% FBS and stained with allophycocyanin-conjugated anti–human DPP4 antibody (BioLegend, cat. #302709) and allophycocyanin-conjugated anti–mouse DPP4 antibody (BioLegend, cat. #137807) or a phycoerythrin-conjugated anti–human PD-L1 antibody (BioLegend, cat. #329738) for 30 minutes at room temperature, washed with Dulbecco’s PBS containing 3% FBS, and analyzed using FACS with FACSAria II (BD Biosciences) or Cell Sorter SH800S (Sony). Allophycocyanin-conjugated mouse IgG2a antibody (BioLegend, cat. #400208), allophycocyanin-conjugated rat IgG2a (BioLegend, cat. #400511), or phycoerythrin-conjugated mouse IgG2b (BioLegend, cat. #400338) was used as an isotype control antibody.

### DPP4 activity assay

Cancer cells were seeded at 3 to 5 × 10^4^ cells per well in 96-well plates and incubated for 24 hours. When indicated, cells were treated with the respective reagents and further incubated for an additional 24 to 48 hours. The culture medium was then replaced with RPMI-1640 without FBS, and supernatants were collected after 6 hours. Aliquots of the collected supernatants were mixed with the DPPIV-Glo substrate/detection reagent and incubated at room temperature to allow enzymatic cleavage of the luminogenic substrate. DPP4 enzymatic activity was quantified using the DPPIV-Glo Protease Assay (Promega, cat. #G8350) according to the manufacturer’s instructions.

### RNA sequencing for cancer cell line analysis and pathway analysis

RNA sequence library preparation, sequencing, mapping, gene expression analysis, and gene ontology enrichment were performed using the DNAFORM software. Total RNA quality was assessed using a bioanalyzer (Agilent) to ensure that RNA integrity was >7. After poly (A) + RNA enrichment using the NEBNext Poly(A) mRNA Magnetic Isolation Module (New England BioLabs), double-stranded cDNA libraries [RNA sequencing (RNA-seq) libraries] were prepared using the SMARTer stranded RNA-Seq kit v2 (Clontech) and the DNBSEQ MGIEasy Universal Library Conversion Kit (MGI Tech), according to the manufacturer’s instructions.

RNA-seq libraries were sequenced using paired-end reads (150 nt of reads 1 and 2) on a DNBSEQ-G400RS instrument (MGI Tech). The obtained raw reads were trimmed and quality-filtered using Trim Galore! (version 0.4.4), Trimmomatic (version 0.36), and Cutadapt (version 1.16). Trimmed reads were mapped to the human GRCh38 genome using STAR (version 2.7.2b). Reads on annotated genes were counted using featureCounts (version 1.6.1). Gene set enrichment analysis (GSEA) was performed using the preranked gene list derived from RNA-seq data. The Molecular Signatures Database ontology gene set was used for the analysis.

### ELISA

Human granzyme B (R&D Systems, DGZB00) ELISAs were performed according to the manufacturer’s instructions. Conditioned media were collected after culturing with H2122 and NK-92 cells for 96 hours and then used for ELISA. Values are the average of three replicates.

### Generation of H2122-specific T cells

Human mononuclear cells from peripheral blood (ultra-pure, single donor; PromoCell; Takara Bio, cat. #C-12907) were used as the peripheral blood mononuclear cell (PBMC) source. To generate H2122-specific T cells, PBMCs were cocultured with mitomycin C (MMC)–treated H2122 cells in AIM-V medium (Invitrogen) supplemented with 3% human male AB serum (Innovative Research), 10 IU/mL IL2, and 10 ng/mL IL15. MMC-treated H2122 cells were added to the culture weekly to expand and maintain the H2122-specific T cells.

### Immune cell migration assay

Immune cell migration assays were performed as previously described (7). Briefly, cancer cell (H2122-GFP or H2009) spheroids were generated by seeding 1 × 10^6^ cells in suspension in an ultralow attachment dish (Thermo Fisher Scientific, cat. #174930) for 24 hours. H2009 cells were stained with Cell Green dye (Invitrogen, cat. #V22886) and spheroids were resuspended in type I collagen according to the manufacturer’s instructions (cat. #638-00781). The spheroid–collagen suspension was then injected into the central gel region of a DAX-1 three-dimensional (3D) microfluidic cell culture chip (AIM Biotech, cat. #DAX-1). NK-92– or H2122-specific T cells were stained with Cell Red dye (Invitrogen, cat. #V22885) to visualize cell migration under a confocal microscope. Cells were cultured for 3 days without human IL2 supplementation, followed by culturing in complete media for 2 days. The treated cells were imaged using an Olympus FV 1000-D or ZEISS LSM 900 microscope with a 10× or 20× objective. Each image was captured using a z-stack with 7.89-μm intervals to encompass the entire cell of interest. All samples were imaged and analyzed using the same settings throughout the experiments.

### Murine and tumor implantation studies

All mouse experiments were conducted in accordance with a protocol approved by Asahikawa Medical University. Five million cells [393P-KL and 393P-DPP4 overexpressing (OE)] in PBS were subcutaneously injected into the dorsal region of 8-week-old male 129-Elite mice (129S2/SvPasCrl, strain code 476, Charles River Laboratories). Five million cells (393P-KL and 393P-DPP4 OE) in PBS were subcutaneously injected into the dorsal region of 8-week-old male NOD/SCID gamma (NSG) mice (NOD.Cg-Prkdcscid Il2rgtm1Wjl/SzJ). In addition, 2 × 10^6^ MC38 colon carcinoma cells were injected subcutaneously into the left flank of 8- to 12-week-old female wild-type C57BL/6JJcl mice. Tumor volume was determined using caliper measurements of tumor length (L) and width (W), according to the formula (L × W^2^)/2. Animals were randomized into various treatment groups every 4 days after cell seeding, before treatment initiation. Observations were conducted for 40 days, and mice with tumor volumes exceeding 2,000 mm^3^ were euthanized. The tumor size was measured twice per week. Anti–mouse PD-1 antibody (clone RMP1-14 from Bio X Cell, cat. #BE0146) and an IgG2a isotype control (clone 2A3 from Bio X Cell, cat. #BE0089) were dissolved in dilution buffer (Bio X Cell, cat. #IP0070) and administered intraperitoneally. In the 393P-KL model, antibodies were administered at a dosage of 200 μg/mouse twice a week for a total of four injections. In contrast, in the MC38 model, antibodies were administered at a dosage of 100 μg/mouse twice a week for a total of three injections. For the CD8 depletion study, tumor-bearing mice were injected intraperitoneally with a CD8^+^ T cell–depleting antibody (clone 53-6.7 from Bio X Cell) diluted in PBS at a concentration of 250 μg/mouse. In satellite animals, tumor tissue was isolated 24 hours after the last treatment and subjected to FACS analysis.

### Immune profiling by flow cytometry

Fresh tumor tissue samples were placed in a dissociation buffer consisting of RPMI (Life Technologies) + 10% FBS (HyClone), 100 U/mL collagenase type IV (Life Technologies), and 50 mg/mL DNase I (Roche) at a ratio of 5 mL of dissociation buffer per 500 mg of sample and mechanically separated using gentleMACS C Tubes and gentleMACS Octo Dissociator system according to the manufacturer’s protocol (Miltenyi Biotec). The suspension was incubated at 37°C for 45 minutes. Red blood cells were removed from the samples using red blood cell lysis buffer (BioLegend). The samples were pelleted and resuspended in fresh RPMI + 10% FBS and strained using a 70-mm filter. Cells were incubated for 5 minutes with Live/Dead Fixable Zombie NIR (BioLegend) in PBS at room temperature in the dark. Fc receptors were blocked using mouse TruStain FcX blocking reagent (BioLegend) before staining with surface antibodies. Cells were stained with preconjugated antibodies for 15 minutes on ice in 2% FBS and washed before analysis using a BD LSRFortessa with FACSDiva software (BD Biosciences). Data were analyzed using FlowJo software version 10.7.1. Antibodies were specific for the following mouse markers: CD3 (17A2), CD4 (GK1.5), CD8 (53-6.7), CD11b (M1/70), CD19 (6D5), CD25 (PC61), CD45 (30-F11), CD49b (DX5), Ly6G (1A8), LAG-3 (C9B7W), PD-1 (29F.1A12), and TIM-3 (B8.2C12) from BioLegend and Foxp3 (FJK-16s) from Thermo Fisher Scientific.

### Statistical analysis

Statistical significance was assessed using an unpaired two-tailed Student *t* test, one-way ANOVA, followed by Tukey *post hoc* test, or two-way ANOVA, followed by Tukey *post hoc* test. Statistical significance was set at *P* < 0.05. Asterisks indicate significance at *, *P* < 0.05; **, *P* < 0.005, and ***, *P* < 0.001. Columns represent means ± SD. In one-way or two-way ANOVA followed by *post hoc* tests, asterisks are shown only in pairs of interest. GraphPad Prism 7 was used for all statistical analyses.

### Data analysis

Publicly available transcriptomic data (OmicsExpressionProteinCodingGenesTPMLogp1) and model annotation (Model.csv) were obtained from the DepMap Public 24Q4 dataset, which contains gene expression profiles of cancer cell lines from the Cancer Cell Line Encyclopedia (CCLE). LKB1-mutant (H1395, H838, H1437, and H1755) and LKB1 wild-type (H2228, H3255, H2087, H1793, and H1650) NSCLC cell lines were selected. Thirty genes, including *DPP4* and *CST6*, previously identified as top differentially expressed genes between KL and KP subtypes, were analyzed. Log1p (transcripts per million) values were *z*-scored and visualized as heatmaps using ComplexHeatmap without clustering. Expression differences between LKB1-mutant and wild-type groups were further evaluated using violin/box plots (ggplot2) and Wilcoxon rank-sum tests with Benjamini–Hochberg FDR correction. In addition, DPP4 expression in The Cancer Genome Atlas Lung Adenocarcinoma dataset (accessed via cBioPortal, last updated in 2018) was analyzed to confirm consistent downregulation in LKB1-mutant tumors.

### Ethics approval

The study protocol was approved by the institutional board of Asahikawa Medical University (approval number: 21117). The study was conducted in accordance with the principles of the Declaration of Helsinki.

## Results

### DPP4 expression is downregulated in KRAS-LKB1 lung cancer

**Figure 1.**
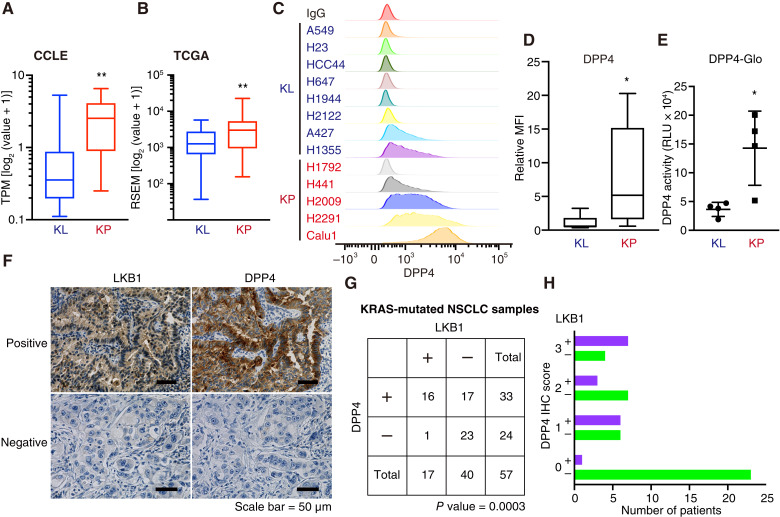
DPP4 expression is downregulated in *KRAS*-*LKB1* lung cancer. **A,** Relative reads per kilobase of transcript per million (TPM) reads mapped (RPKM) values of DPP4 in KL (blue) and KP (red) cells derived from Cancer Cell Line Encyclopedia mice. **B,** Relative RPKM values of DPP4 in the KL (blue) and KP (red) samples from The Cancer Genome Atlas (TCGA). **C,** DPP4 expression on the surface of KL (blue) and KP (red) cells. **D,** Mean fluorescence intensity (MFI) of DPP4 expression was quantified using FlowJo. **E,** DPP4 activity induced by DPP4-Glo in KL (blue) and KP (red) cells. **F,** Representative DPP4 and LKB1 IHC images of *KRAS*-mutated NSCLC samples. **G** and **H,** DPP4 and LKB1 expression in *KRAS*-mutated NSCLC samples. DPP4 staining intensity was scored in a blinded manner on a scale of 0–3: IHC0, no staining; IHC1, weak staining; IHC2, moderate staining; IHC3, strong staining. IHC1–3 was defined as positive DPP4 expression, and IHC0 was defined as negative DPP4 expression. *P* values were calculated using an unpaired two-tailed Student’s *t *test (**A, B,D,** and** E**) or Fisher’s exact test (**G**). *, *P* < 0.05; **, *P* < 0.01. RLU, relative light unit; RSEM, RNA-Seq by Expectation Maximization.

We previously compared the genomic profiles of KL and KP cells to identify potential molecular targets (10). Among the downregulated molecules identified, DPP4 emerged as a gene of particular interest because of its marked suppression in KL cells compared with KP cells. To validate these findings, we reanalyzed DPP4 expression using the updated DepMap Public 24Q4 dataset, released in 2024 and derived from the CCLE. Consistently, transcriptomic analysis comparing LKB1-mutant and wild-type NSCLC cell lines within the CCLE dataset demonstrated that DPP4, along with several previously reported KL-downregulated genes, was selectively suppressed in the LKB1-mutant group (Supplementary Fig. S1A and S1B). DPP4 expression was also analyzed using the Lung Adenocarcinoma dataset, which was last updated in 2018 and accessed via cBioPortal, referred to as The Cancer Genome Atlas. These analyses revealed that DPP4 expression was significantly lower in KL cells than in KP cells (Fig. 1A and B). Subsequently, we examined DPP4 expression in *KRAS*-mutant lung cancer cell lines. DPP4 cell surface expression was significantly downregulated in KL cells compared with that in KP cells (Fig. 1C and D; Supplementary Fig. S1C). We further validated these results by performing a functional analysis using the DPP4-Glo assay, which confirmed that DPP4 activity was lower in KL cells than in KP cells (Fig. 1E).

We also assessed tumor cell–specific DPP4 and LKB1 protein levels using IHC in a panel of 57 patient-derived NSCLC samples, which were enriched for *KRAS* mutations. LKB1 loss strongly correlated with a reduction in DPP4 levels in tumor cells (*P* = 0.0364; Fig. 1F–H). These findings suggest a significant correlation between LKB1 loss and the downregulation of DPP4 in *KRAS*-mutant lung cancer.

### LKB1 modulates DPP4 expression in KRAS-mutant lung cancer

**Figure 2.**
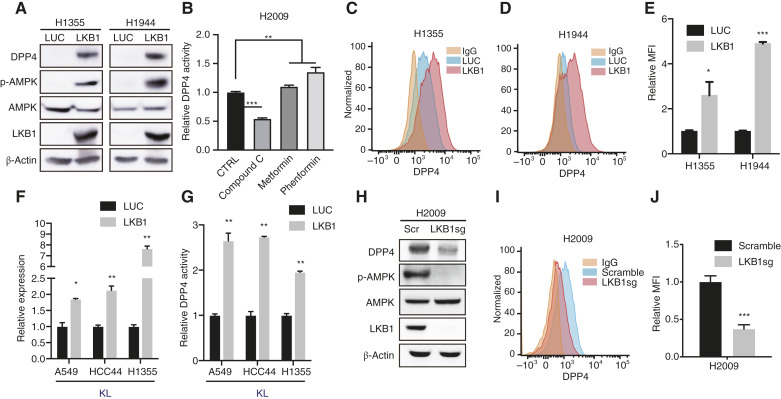
LKB1 modulates DPP4 expression in *KRAS*-mutant lung cancer. **A,** Immunoblotting of the indicated proteins in KL cells transduced with the indicated vectors. **B,** DPP4 activity measured by DPP4-Glo in H2009 KP cells treated with 10 μmol/L compound C, 10 μmol/L metformin, or 200 μmol/L phenformin (*n* = 3) for 24 hours. **C** and **D, **DPP4 expression on the surface of H1355 and H1944 KL cells transfected with the indicated vectors. **E,** MFI of DPP4 expression in KL cells transfected with the indicated vectors, quantified using FlowJo (*n* = 3). **F,** qRT-PCR analysis of *DPP4* expression in KL cells transfected with the indicated vectors (*n* = 2). **G,** DPP4 activity of DPP4-Glo in KL cells transfected with the indicated vectors (*n* = 3). **H,** Immunoblotting of the indicated proteins in H2009 KP cells transduced with the indicated vectors. **I, **DPP4 expression on the surface of H2009 KP cells transfected with the indicated vectors. **J,** MFI of DPP4 expression in H2009 KP cells transfected with the indicated vectors, quantified using FlowJo (*n* = 3). *P* values were calculated using an unpaired two-tailed Student's *t *test. *, *P* < 0.05; **, *P* < 0.01; ***, *P* < 0.001.

We then investigated whether LKB1 directly regulates DPP4 expression. LKB1 reconstitution in KL cells (H1355 and H1944), which were selected as representative KL cell lines owing to their lack of LKB1 expression, promoted AMPK phosphorylation (Fig. 2A; Supplementary Fig. S2A). This phosphorylation was accompanied by the upregulation of DPP4 expression, suggesting a link between LKB1–AMPK signaling and DPP4 expression (Fig. 2A). Subsequently, we treated H2009 cells with metformin and phenformin, both AMPK activators that markedly increased DPP4 activity. In contrast, treatment with compound C, an AMPK inhibitor, significantly reduced DPP4 activity (Fig. 2B). The corresponding changes in phospho-AMPK expression were confirmed by Western blot analysis (Supplementary Fig. S2B). We performed a FACS analysis to assess whether the increase in DPP4 expression observed in H1355 and H1944 cells with LKB1 reconstitution was localized to the cell surface. A significant increase in DPP4 expression was observed on the cell surface, indicating membrane localization (Fig. 2C–E). Restoration of DPP4 expression following LKB1 reconstitution was further validated at the mRNA level using qRT-PCR. DPP4 functional activation was observed in KL cells (A549, HCC44, and H1355; Fig. 2F and G). Furthermore, CRISPR/Cas9-mediated depletion of LKB1 in KP cells (H2009 and H1792), which were selected because of their elevated LKB1 expression levels, resulted in a reduction in DPP4 mRNA levels and functional activity (Supplementary Fig. S2C and S2D). This effect was further validated at the protein level in H2009 cells, with subsequent quantification confirming a corresponding decrease in DPP4 surface levels (Fig. 2H–J). Importantly, a similar reduction in DPP4 expression was observed when LKB1 was depleted in the KRAS wild-type NSCLC cell line H1793, further indicating that DPP4 downregulation is primarily driven by LKB1 loss rather than KRAS mutation status (Supplementary Fig. S2E). Overall, these data suggest that LKB1 directly regulates DPP4 expression in *KRAS*-mutant NSCLC cells.

### DPP4 as a potential therapeutic target in KRAS-mutant cells via NK-cell recruitment

**Figure 3.**
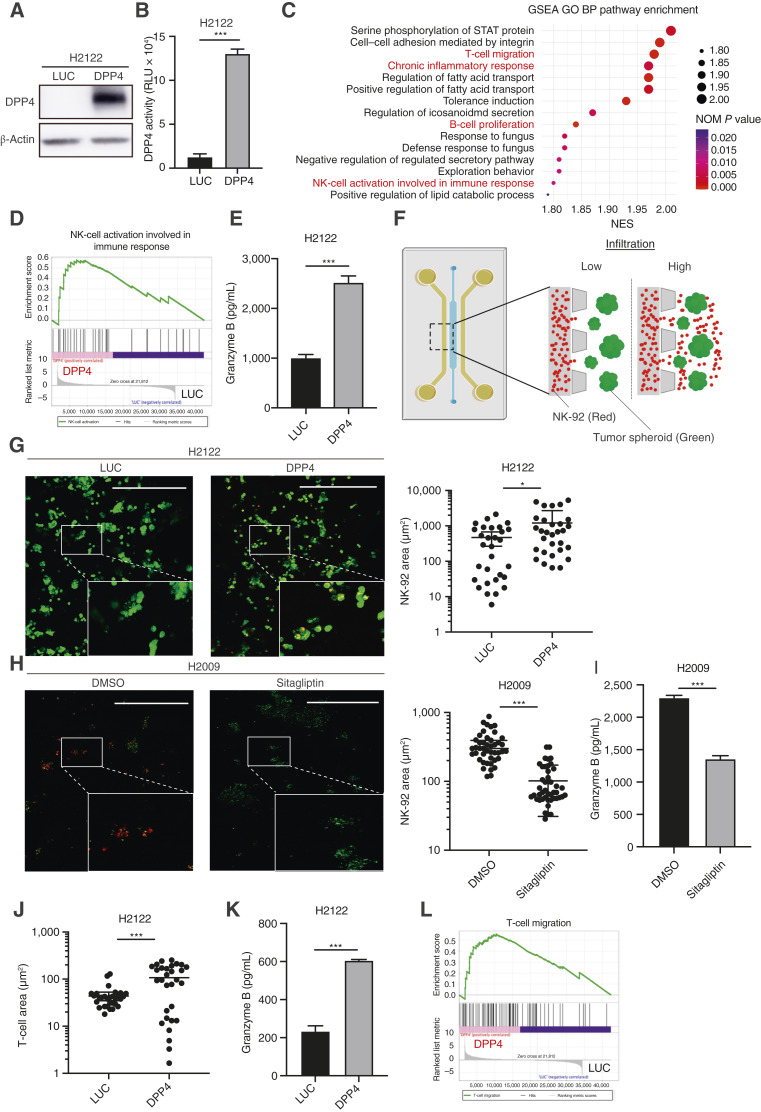
DPP4 is a potential therapeutic target in *KRAS*-mutant cells via NK cell recruitment. **A,** Immunoblotting of the indicated proteins in KL H2122 cells transduced with the indicated vectors. **B,** DPP4 activity induced by DPP4-Glo in KL H2122 cells transduced with the indicated vectors (*n* = 3). **C,** GSEA showing significantly differentially expressed pathways. GSEA Gene Ontology Biological Process (GO BP) category upregulation in DPP4-reconstituted H2122 cells relative to luciferase (LUC)-reconstituted H2122 cells. **D,** GSEA of NK cell activation involved in the immune response signature in H2122 cells transduced with DPP4 or LUC. **E,** ELISA for human granzyme B in conditioned medium (CM) derived from H2122 cells cocultured with NK-92 cells (*n* = 3). **F,** Schematic of an immune cell migration assay using a three-dimensional microfluidic device with tumor spheroids embedded in a central collagen-filled channel and immune cells cocultured in a side channel. **G,** Representative images of NK-92 cell migration toward H2122 or DPP4-overexpressing H2122 cells (left). Immune infiltration into the peritumor region was quantified using ImageJ software (right). Scale bars, 500 μm. **H,** Representative images of NK-92 cell migration in KP H2009 cells treated with and without sitagliptin (left). Immune infiltration into the peritumor region was quantified using ImageJ software (right). Scale bars, 500 μm. **I,** ELISA for human granzyme B in CM derived from H2009 cells treated with and without sitagliptin and cocultured with NK-92 cells (*n* = 3). **J,** Evaluation of H2122-specific CD8^+^ T-cell migration toward H2122 or DPP4-overexpressing H2122 cells. Immune infiltration into the peritumor region was quantified using ImageJ software. **K,** ELISA for human granzyme B in CM derived from H2122 cells cocultured with H2122-specific CD8^+^ T cells (*n* = 3). **L,** GSEA of T-cell migration involved in the immune response signature in H2122 cells transduced with DPP4 or LUC. *P* values were calculated using an unpaired two-tailed Student's *t *test. *, *P* < 0.05; ***, *P* < 0.001.

The potential of DPP4 as a therapeutic target in KL cells was investigated. Reconstituted DPP4 in KL cell lines (H2122 and H1944) increased DPP4 expression and functional activity (Fig. 3A and B; Supplementary Fig. S3A and S3B). However, DPP4 reconstitution did not affect the proliferation of KL cells under two-dimensional culture conditions (Supplementary Fig. S3C and S3D). RNA-seq was conducted to compare the transcriptomic profiles of DPP4-reconstituted H2122 cells and investigate the impact of DPP4 reconstitution on gene expression. GSEA revealed that DPP4 reconstitution significantly induced multiple immune-related signatures, including chronic inflammatory responses and B-cell proliferation (Fig. 3C; Supplementary Fig. S3E and S3F). These findings indicate that DPP4 may play a role in antitumor immunity. Among the identified signatures, NK-cell activation, which is involved in the immune response, was significantly enriched following DPP4 reconstitution (Fig. 3C and D). The conditioned medium from the two-dimensional coculture of H2122 or H1944 cells with NK-92 cells showed increased granzyme B levels following DPP4 reconstitution (Fig. 3E; Supplementary Fig. S3G), indicating enhanced NK-cell activity. Based on these observations, we examined NK-cell migration in response to DPP4 reconstitution using a previously described 3D microfluidic system (Fig. 3F; ref. 7). DPP4-reconstituted H2122 cells significantly accelerated the migration of NK-92 cells from the side channels toward the tumor spheroids (Fig. 3G). Conversely, the pharmacologic inhibition of DPP4 attenuated NK-cell migration. The treatment of the KP cell line H2009 with sitagliptin reduced NK-92 cell migration, and sitagliptin treatment of DPP4-OE H2122 cells similarly diminished NK migration compared with untreated controls (Fig. 3H and I; Supplementary Fig. S3H). Consistently, sitagliptin treatment markedly suppressed DPP4 enzymatic activity, as validated by the functional assay (Supplementary Fig. S3I and S3J), further supporting that NK-cell migration is closely linked to DPP4 enzymatic function. To complement the NK-cell assays, we generated H2122-specific CD8^+^ T cells from PBMCs and assessed their migratory response using the same 3D microfluidic system. The DPP4-reconstituted H2122 cells significantly accelerated the migration of CD8^+^ T cells from the side channels toward the tumor spheroids, similar to the effects observed with NK-92 cells (Fig. 3J and K). This is consistent with the GSEA results in which T-cell migration was enriched after the reconstitution of DPP4 (Fig. 3C and L).

These results demonstrate that DPP4 expression in KL cells enhances both NK and CD8^+^ T-cell recruitment and activation, thereby reshaping the tumor–immune microenvironment. These findings highlight the potential of DPP4 as a therapeutic strategy for overcoming immune resistance in *KRAS-LKB1*–mutant lung cancer.

### Overexpression of DPP4 promotes tumor regression in syngeneic KL model *in vivo*

The use of DPP4 as a therapeutic target in immunotherapy was initially proposed based on *in vitro* studies using human KL lung cancer cell lines. In this study, a syngeneic murine 393P-KL model was used to investigate whether DPP4 reconstitution enhances ICI sensitivity (8).

**Figure 4.**
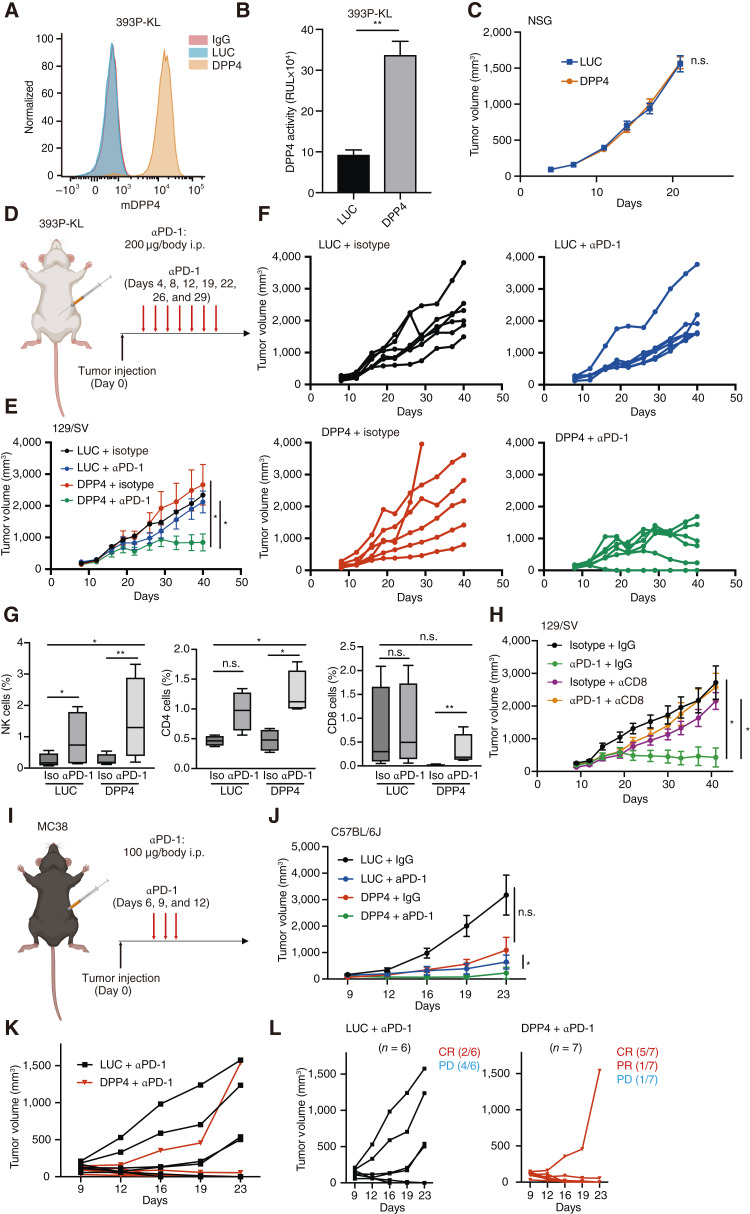
DPP4 overexpression promotes tumor regression in the syngeneic KL model *in vivo*. **A,** mDPP4 expression on the surface of 393P-KL cells transfected with the indicated vectors. **B,** DPP4 activity of DPP4-Glo in KL cells transfected with the indicated vectors. **C,** Mean tumor volume of 393P-KL cells after subcutaneous inoculation in NSG mice. n.s., not significant (unpaired two-tailed Student’s *t *test). **D,** Schematic of the efficacy study with anti–PD-1 antibody in a synergistic murine KL model. **E,** Mean tumor volume of 393P-KL cells after subcutaneous inoculation into 129S2/SvPasCrl mice. **F,** Tumor volume of 393P-KL cells after subcutaneous inoculation into 129S2/SvPasCrl mice, followed by anti–PD-1 antibody on days 4, 8, 12, 19, 22, 26, and 29. **G,** NK (left), CD4 (middle), and CD8 (right) cells are shown as percentages in all groups (*n* = 4). **H,** Mean tumor volume of DPP4-overexpressing 393P-KL cells after subcutaneous inoculation into 129S2/SvPasCrl mice, evaluated after treatment with anti-CD8 neutralization antibody, anti–PD-1 antibody, both antibodies, or no treatment. **I,** Schematic for the efficacy study using an anti–PD-1 antibody in a synergistic murine model. **J,** Mean tumor volume of MC38 cells after subcutaneous inoculation into C57BL/6 mice.** K,** Tumor volume of MC38 cells after subcutaneous inoculation into C57BL/6 mice, followed by anti–PD-1 antibody on days 6, 9, and 12. **L,** Antitumor effect of anti–PD-1 in the MC38 syngeneic model. Spider plots show individual tumor growth in C57BL/6 mice treated with anti–PD-1. *P* values were calculated using an unpaired two-tailed Student *t *test. *, *P* <0.05; **, *P* < 0.01.

DPP4 was not expressed in the parental 393P-KL cell line (Fig. 4A). We then generated DPP4-reconstituted 393P-KL cell lines, which showed restored enzymatic activity, to investigate whether increased DPP4 expression influenced the immune system (Fig. 4A and B). No significant differences in tumor growth were observed between DPP4-reconstituted cells and control cells in two-dimensional cultures, suggesting that DPP4 expression alone did not affect intrinsic tumor growth *in vitro* (Supplementary Fig. S4A). Immunodeficient NSG mice were used to examine whether DPP4 influences tumor growth *in vivo*. However, no significant differences in tumor growth were observed between the DPP4-reconstituted and control 393P-KL cell lines in this model, which was consistent with the *in vitro* results (Fig. 4C). Immunocompetent 129/SvPasCrl mice were used to assess the impact of DPP4 in the context of an intact immune system to investigate the immunotherapeutic implications of DPP4. Specifically, we tested whether DPP4 activation enhanced sensitivity to anti–PD-1 therapy (Fig. 4D). No significant differences in tumor growth were observed between DPP4-reconstituted and control cells in the absence of anti–PD-1 treatment. However, tumors derived from DPP4-reconstituted 393P-KL cells exhibited significantly reduced growth compared with those derived from control cells upon anti–PD-1 treatment (Fig. 4E and F). Collectively, these findings suggest that DPP4 activation plays a crucial role in enhancing the response to anti–PD-1 therapy in a fully intact immune environment. Consistent with these findings, the analysis of tumor-infiltrating lymphocytes revealed an increase in NK cells and CD4^+^ T cells in the DPP4-reconstituted group compared with controls, which is consistent with the enhanced *in vivo* antitumor effect. Although overall CD8^+^ T-cell infiltration did not differ significantly between the control and DPP4-reconstituted tumors, anti–PD-1 treatment led to a marked increase in CD8^+^ T-cell infiltration in the DPP4-reconstituted group (Fig. 4G). In addition, RNA analysis of resected tumors without ICI treatment revealed an increasing trend in the expression of multiple immune-related markers, including CD4, CD8, CXCL10, granzyme B, TNFα, and IFNβ, in the DPP4-reconstituted group compared with the control group. However, statistical significance was not reached (Supplementary Fig. S4B–S4G). Consistent with a key role for CD8^+^ T-cell immunity, the antitumor effect of anti–PD-1 therapy in the DPP4-reconstituted group was abolished by CD8^+^ T-cell depletion, resulting in renewed tumor growth (Fig. 4H; Supplementary Fig. S4H). The antitumor effects of DPP4 in combination with ICI therapy were further evaluated using an additional syngeneic mouse model. Specifically, we tested whether DPP4 reconstitution enhances sensitivity to ICIs in the MC38 colorectal cancer cell line, which does not harbor KRAS mutations. As with the 393P-KL cells, no significant differences in tumor growth were observed in two-dimensional cultures between DPP4-reconstituted MC38 cells and control cells, indicating that DPP4 expression alone did not affect intrinsic tumor growth *in vitro* (Supplementary Fig. S4E). Consistent with the findings in the 393P-KL model, DPP4-reconstituted MC38 tumors showed enhanced antitumor responses to anti–PD-1 treatment (Fig. 4I and J). A detailed analysis of the PD-1–treated groups revealed that two of six mice in the control cohort achieved a complete response (CR), and four exhibited progressive disease. In contrast, five of seven mice in the DPP4-reconstituted group achieved CR, one showed a partial response, and one showed disease progression (Fig. 4K and L). These findings further support the involvement of DPP4 in antitumor immunity *in vivo*.

## Discussion

This study provides the first evidence that the LKB1 tumor suppressor gene actively regulates the expression of DPP4, which is a pivotal immune modulator in KRAS-mutated NSCLC. In this challenging subset of KRAS-driven lung cancers, the loss of LKB1 leads to a profound immunosuppressive TME characterized by diminished immune cell infiltration and reduced sensitivity to ICIs (4). This downregulation of DPP4 represents an active mechanism of immune evasion, by which tumor cells exploit LKB1 deficiency to suppress pathways critical for immune activation. Our findings are in line with recent clinical observations showing that *STK11* and *KEAP1* co-mutations are significantly associated with “desert” or PD-L1–negative immune phenotypes in NSCLC, supporting the link between LKB1 loss, low DPP4 expression, and an immune-cold TME (11). Moreover, our study demonstrated that the restoration of DPP4 expression reprogrammed the TME, enhanced immune effector cell recruitment, and significantly improved responsiveness to PD-1 blockade therapy. These findings establish DPP4 as a central player in the immune landscape of KL-NSCLC, highlighting its potential as a novel therapeutic target to overcome ICI resistance.

DPP4, also known as CD26, is a dimeric transmembrane glycoprotein with a maximum molecular weight of 110 kDa, which is expressed in various cell types, including epithelial cells and lymphocytes. Initially identified as an adenosine deaminase–binding protein, DPP4 was found to cleave N-terminal X-proline or X-alanine dipeptides from polypeptides, thereby playing a role in protein digestion (9, 12). In addition to its enzymatic function, DPP4 interacts with extracellular matrix components, including collagen and fibrinogen, as well as cell surface proteins such as CD45, caveolin-1, and β-integrins. These diverse functions position DPP4 as a central player in cellular communication and immune regulation (13).

DPP4 plays a dual role in modulating antitumor immunity by acting as both a promoter and suppressor of immune responses, depending on the tumor context and immune microenvironment (9). For example, Jang and colleagues (14) reported that DPP4 inhibition suppressed lung cancer growth via macrophage–NK-cell interactions, with increased DPP4 expression observed in patients with NSCLC, further highlighting the context-dependent roles of DPP4. In specific settings, DPP4 inhibitors enhance antitumor immunity by preserving the functional integrity of chemokines, such as CXCL10 (15), whereas DPP4 inhibition sustains the ability of CXCL10 to recruit CXCR3-expressing lymphocytes to the tumor parenchyma by preventing its enzymatic truncation, thereby improving immune cell trafficking and amplifying the efficacy of immunotherapies, including ICIs. Consistent with these context-dependent effects, Zuo and colleagues (16) demonstrated that the DPP4 inhibitor anagliptin could be synergized with anti–PD-L1 therapy, as it reprogrammed tumor-associated macrophages, further underscoring that DPP4 function can vary with tumor context.

Our study presents a novel perspective by demonstrating that restoring DPP4 expression, rather than inhibiting it, reprograms the TME to promote antitumor immunity. DPP4 restoration enhances the recruitment of immune effector cells, particularly NK-cell migration, in KL tumors. Although the mechanisms remain unclear, these findings suggest that DPP4 is involved in modulating the adenosine pathway, indicating that further investigation is required to elucidate its impact on the immune microenvironment (17). Notably, the observed increase in NK-cell migration was consistent with the potential role of DPP4 in shaping a more permissive immune microenvironment even though we did not directly investigate adenosine metabolism. These results provide a basis for further studies to examine the involvement of DPP4 in NK-cell recruitment and the adenosine pathway.

Our *in vivo* syngeneic KL model demonstrated a combinatory effect of anti–PD-1 treatment when administered to DPP4-reconstituted 393P-KL cell lines. Although DPP4 restoration alone did not result in a significant therapeutic effect, this might be partially explained by the interaction between CD26 and other immunosuppressive factors, such as CD73 (18). Nonetheless, the critical finding is that DPP4 restoration contributed to the activation of the immune system, which in turn enhanced the efficacy of anti–PD-1 treatment. Moreover, tumor regression requires the presence of DPP4-reconstituted elements, underscoring the indispensable role of these elements. Although much attention has been paid to the enzymatic function of DPP4, its nonenzymatic role impairs regulatory T-cell (Treg) function. Specifically, DPP4 interacts with insulin-like growth factor II on the surface of Tregs, resulting in the functional suppression of these immunosuppressive cells (19). This mechanism highlights the multifaceted contributions of DPP4 in modulating the immune landscape and enhancing the responses to immune checkpoint blockade. The randomized phase III POSEIDON trial reported the potential of immune combination therapy using CTLA4 and PD-(L)1 inhibitors to overcome ICI resistance in KL (20). Although this clinical observation is independent of our study, it is conceivable that the CTLA4 blockade remodels the TME in ways that could indirectly influence DPP4 pathways. Although speculative, this highlights an interesting direction for future research.

Nevertheless, this study has several limitations. First, although metabolic changes characteristic of LKB1 mutations have been reported (21–23), their relationship with DPP4 functions remains unclear. Moreover, the precise mechanism by which DPP4 reprograms the TME is also unknown. Although our findings demonstrated that DPP4 restoration enhanced immune cell recruitment and responsiveness to PD-1 blockade, the pathways mediating these effects remain to be clarified. Further investigation is warranted to elucidate how DPP4 functionally shapes the tumor–immune microenvironment. Second, this study clearly demonstrates that LKB1 loss is associated with reduced DPP4 expression in KRAS-mutant NSCLC. However, the relationship between DPP4 and other representative resistance-associated co-mutations, such as *KEAP1*, remains to be clarified. Further investigations are required to determine whether these additional co-mutations influence DPP4 regulation or contribute independently to immune resistance. Finally, our findings also showed that the AMPK activator metformin effectively increased DPP4 activity, suggesting that pharmacologic agents capable of inducing or activating DPP4 may enhance the immunogenicity of the TME. Future studies should explore the potential of the DPP4 activator as a therapeutic approach to reprogram the TME and improve the response to ICIs. Recently, DPP4 has garnered attention as a potential biomarker (24). However, further research is required to elucidate its role as a biomarker for ICIs in *KRAS*-mutant lung cancer and its relationship with other co-mutations associated with ICI resistance.

In conclusion, our results suggest that DPP4 is a promising target for overcoming ICI resistance in *KRAS-LKB1*–mutant NSCLC cells. We demonstrated a significant enhancement in immune cell recruitment and response to PD-1 blockade by restoring DPP4 expression, providing a strong foundation for the future exploration of DPP4-directed therapies. These findings advance our understanding of the immune resistance mechanisms in KL-NSCLC and highlight the potential of DPP4-based interventions to redefine the therapeutic landscape of this challenging subset of lung cancer.

## Supplementary Material

Table S1 Table S1: The sgRNA target sequences

Figure S1 DPP4 expression is downregulated in KRAS-LKB1 lung cancer.

Figure S2 LKB1 modulates DPP4 expression in KRAS-mutant lung cancer.

Figure S3 DPP4 is a potential therapeutic target in KRAS-mutant cells due to NK cell recruitment.

Figure S4 DPP4 overexpression promotes tumor regression in the syngeneic KL model in vitro.

## Data Availability

The datasets generated and analyzed in this study are available upon reasonable request from the corresponding author. The raw and processed data have been deposited in the Gene Expression Omnibus under accession number GSE302247.
