# Supplementary material for: Dipeptidyl Peptidase 4 Restoration Facilitates Antitumor Immunity in KRAS-LKB1–Mutant Lung Cancer
Source: Cancer Res Commun. 2025 Dec 17;5(12):2175–85. doi: 10.1158/2767-9764.CRC-25-0199 (PMC12709056; doi:10.1158/2767-9764.CRC-25-0199)
Supplement: Figure S1 — DPP4 expression is downregulated in KRAS-LKB1 lung cancer. [file crc-25-0199_figure_s1_suppsf1.docx]

**Supplementary Figure S1. DPP4 expression is downregulated in *KRAS*-*LKB1* lung cancer.**

**A**.  Heatmap showing the expression levels (Z-score) of 30 representative genes, including DPP4, previously identified as differentially expressed between KL (LKB1-mutant) and KP (LKB1-wild-type) subtypes in the CCLE dataset. **B**. Violin and box plots comparing DPP4 transcript levels between LKB1-mutant and wild-type NSCLC cell lines based on the DepMap Public 24Q4 dataset. **C**. qRT-PCR analysis of *DPP4* expression in KL and KP cells.
