# Supplementary material for: Dipeptidyl Peptidase 4 Restoration Facilitates Antitumor Immunity in KRAS-LKB1–Mutant Lung Cancer
Source: Cancer Res Commun. 2025 Dec 17;5(12):2175–85. doi: 10.1158/2767-9764.CRC-25-0199 (PMC12709056; doi:10.1158/2767-9764.CRC-25-0199)
Supplement: Figure S2 — LKB1 modulates DPP4 expression in KRAS-mutant lung cancer. [file crc-25-0199_figure_s2_suppsf2.docx]

**Supplementary Figure S2. LKB1 modulates DPP4 expression in *KRAS*-mutant lung cancer.**

**A.** Immunoblotting of the indicated proteins in KRAS-mutant lung cancer cell lines. **B**. Immunoblotting of the indicated proteins in KP H2009 cells treated with 10 μM of compound C and 10 μM metformin for 24 h. **C**. qRT-PCR analysis of *DPP4* expression in KP cells transfected with scrambled siRNA or LKB1 (n = 2). **D**. DPP4 activity of DPP4-Glo in KL cells transfected with scrambled shRNA or LKB1 (n = 3). **E.** Immunoblotting of the indicated proteins in H1793 cells transfected with the indicated vectors.
