# Supplementary material for: Dipeptidyl Peptidase 4 Restoration Facilitates Antitumor Immunity in KRAS-LKB1–Mutant Lung Cancer
Source: Cancer Res Commun. 2025 Dec 17;5(12):2175–85. doi: 10.1158/2767-9764.CRC-25-0199 (PMC12709056; doi:10.1158/2767-9764.CRC-25-0199)
Supplement: Figure S3 — DPP4 is a potential therapeutic target in KRAS-mutant cells due to NK cell recruitment. [file crc-25-0199_figure_s3_suppsf3.docx]

**Supplementary Figure S3.** **DPP4 is a potential therapeutic target in *KRAS*-mutant cells due to NK cell recruitment.**

**A**. Immunoblotting of the indicated proteins in the KL cell line H1944 transduced with the indicated vectors. **B**. DPP4 activity induced by DPP4-Glo in the KL cell line H1944 transduced with the indicated vectors (n = 3). **C**-**D.** Total number of H2122 and H1944 cells transduced with the indicated vectors at each time point (0, 2, 5, 8, and 12 days; n = 3), respectively. **E**-**F.** GSEA of chronic inflammatory response and the B cell proliferation signature in H2122 cells transduced with DPP4 or LUC. **G.** ELISA of human granzyme B in CM derived from H1944 cells co-cultured with NK-92 cells (n = 3). **H.** Evaluation of NK92 cell migration in DPP4-overexpressing H2122 cells treated or not treated with sitagliptin. **I-J.** DPP4 activity of DPP 4-Glo in H2009 or DPP4-overexpressing H2122 cells treated or not treated with sitagliptin (n = 3).
