# Supplementary material for: Dipeptidyl Peptidase 4 Restoration Facilitates Antitumor Immunity in KRAS-LKB1–Mutant Lung Cancer
Source: Cancer Res Commun. 2025 Dec 17;5(12):2175–85. doi: 10.1158/2767-9764.CRC-25-0199 (PMC12709056; doi:10.1158/2767-9764.CRC-25-0199)
Supplement: Figure S4 — DPP4 overexpression promotes tumor regression in the syngeneic KL model in vitro. [file crc-25-0199_figure_s4_suppsf4.docx]

**Supplementary Figure S4. DPP4 overexpression promotes tumor regression in the syngeneic KL model in vitro.**

**A**. In total, 393P-KL cells were transduced with the indicated vectors at each time point (0, 2, 4, 7, and 13 days; n = 3). **B-G**. qRT-PCR expression analysis of the indicated genes in 393P-KL cells. **H.** Schematic of the efficacy study with anti-PD1 antibody in a synergistic murine KL model. **I**. Total number of MC38 cells transduced with the indicated vectors at each time point (0, 2, 4, and 6 days; n = 3).
